# Typhoid Fever Surveillance, Incidence Estimates, and Progress Toward Typhoid Conjugate Vaccine Introduction — Worldwide, 2018–2022

**DOI:** 10.15585/mmwr.mm7207a2

**Published:** 2023-02-17

**Authors:** Molly Hancuh, Jenny Walldorf, Anna A. Minta, Carol Tevi-Benissan, Kira A. Christian, Yoann Nedelec, Kristen Heitzinger, Matthew Mikoleit, Amanda Tiffany, Adwoa D. Bentsi-Enchill, Lucy Breakwell

**Affiliations:** ^1^CDC Foundation, Atlanta, Georgia; ^2^Department of Immunization, Vaccines, and Biologicals, World Health Organization, Geneva, Switzerland; ^3^Division of Global Health Protection, Center for Global Health, CDC; ^4^Division of Foodborne, Waterborne, and Environmental Diseases, National Center for Emerging, Zoonotic, and Infectious Diseases, CDC; ^5^Global Immunization Division, Center for Global Health, CDC.

Typhoid fever, an acute febrile illness caused by *Salmonella enterica* serovar Typhi (*S*. Typhi), is endemic in many low- and middle-income countries^†^ (*1*). In 2015, an estimated 11–21 million typhoid fever cases and 148,000–161,000 associated deaths occurred worldwide (*2*). Effective prevention strategies include improved access to and use of infrastructure supporting safe water, sanitation, and hygiene (WASH); health education; and vaccination (*1*). The World Health Organization (WHO) recommends programmatic use of typhoid conjugate vaccines for typhoid fever control and prioritization of vaccine introduction in countries with the highest typhoid fever incidence or high prevalence of antimicrobial-resistant *S*. Typhi (*1*). This report describes typhoid fever surveillance, incidence estimates, and the status of typhoid conjugate vaccine introduction during 2018–2022. Because routine surveillance for typhoid fever has low sensitivity, population-based studies have guided estimates of case counts and incidence in 10 countries since 2016 (*3*–*6*). In 2019, an updated modeling study estimated that 9.2 million (95% CI = 5.9–14.1) typhoid fever cases and 110,000 (95% CI = 53,000–191,000) deaths occurred worldwide, with the highest estimated incidence in the WHO South-East Asian (306 cases per 100,000 persons), Eastern Mediterranean (187), and African (111) regions (*7*). Since 2018, five countries (Liberia, Nepal, Pakistan, Samoa [based on self-assessment], and Zimbabwe) with estimated high typhoid fever incidence (≥100 cases per 100,000 population per year) (*8*), high antimicrobial resistance prevalence, or recent outbreaks introduced typhoid conjugate vaccines into their routine immunization programs (*2*). To guide vaccine introduction decisions, countries should consider all available information, including surveillance of laboratory-confirmed cases, population-based and modeling studies, and outbreak reports. Establishing and strengthening typhoid fever surveillance will be important to measure vaccine impact.

## Surveillance and Estimates of Disease Incidence and Antimicrobial Resistance Prevalence

WHO recommends that countries with endemic typhoid fever^§^ establish health facility–based surveillance with laboratory confirmation to determine disease burden,^¶^ monitor antimicrobial resistance patterns, facilitate rapid outbreak detection, and assess vaccine impact (*3*). Because the clinical presentation of typhoid fever is often indistinguishable from that of other acute febrile illnesses common in areas with endemic typhoid (e.g., malaria and dengue), diagnosis is dependent upon laboratory confirmation, typically blood culture (*3*). However, blood culture has a low sensitivity (40%–60%), which is further reduced by widespread use of prediagnosis antibiotic use, has limited availability at health care facilities, and is not systematically obtained from febrile patients (*1*–*3*). Therefore, the number of laboratory-confirmed *S*. Typhi cases represents a small proportion of the actual disease incidence. Countries report data on selected vaccine-preventable diseases to WHO and UNICEF annually using the electronic Joint Reporting Form (eJRF). During 2018–2021, 59–62 countries reported laboratory-confirmed typhoid fever through eJRF.** Reported cases increased from approximately 8,800 in 2018, when typhoid fever surveillance was first added to eJRF, to 1 million in 2021.

Because of the low sensitivity of typhoid fever surveillance, specially designed population-based studies have been implemented to estimate disease incidence. Since 2016, typhoid fever incidence has been estimated in specific countries through three surveillance projects: 1) the Strategic Typhoid Alliance across Africa and Asia (for Bangladesh, Malawi, and Nepal); 2) the Surveillance for Enteric Fever in Asia Project (for Bangladesh, Nepal, and Pakistan); and 3) the Severe Typhoid in Africa program (for Burkina Faso, Democratic Republic of the Congo, Ethiopia, Ghana, Madagascar, and Nigeria) (Table 1) (*4*–*6*). Modeling data from the Global Burden of Disease study estimated that 9.2 million (95% CI = 5.9–14.1) typhoid fever cases and 110,000 (95% CI = 53,000–191,000) associated deaths occurred worldwide in 2019 (*7*). The highest estimated 2019 incidence, by region, occurred in the WHO South-East Asian (306 cases per 100,000 persons), Eastern Mediterranean (187), and African (111) regions (Table 1) (Figure) and, by age group, occurred in children aged 5–9 years, followed by children and adolescents aged 10–14 years and children aged 1–4 years, respectively.^††^

**TABLE 1:** Population-based and modeling estimates of typhoid fever incidence* — worldwide, 2016–2020

Study	Site	Period	Observed no. of cases reported	Incidence* (95% CI)	
				Crude	Adjusted
SEAP^†^	Bangladesh: Dhaka Shishu Hospital and Shishu Shasthya Foundation Hospital	Sep 2016–Sep 2019	4,131	103 (97–109)	913 (765–1095)
	Nepal: Dhulikhel Hospital	Sep 2016–Sep 2019	NA	36 (24–51)	268 (202–362)
	Nepal: Kathmandu Medical College	Sep 2016–Sep 2019	NA	31 (26–37)	330 (230–480)
	Pakistan: Aga Khan University Hospital	Sep 2016–Sep 2019	NA	12 (10–14)	103 (85–126)
	Pakistan: Kharadar General Hospital	Sep 2016–Sep 2019	NA	24 (21–28)	176 (144–216)
STRATAA^§^	Blantyre, Malawi	Nov 2016–Oct 2018	115	58 (48–70)	444 (347–717)
	Kathmandu, Nepal	Jan 2017–Dec 2018	150	74 (62–87)	1,062 (683–1,839)
	Dhaka, Bangladesh	Jan 2017–Dec 2018	359	161 (145–179)	1,135 (898–1,480)
SETA^¶^	Nioko and Polesgo, Burkina Faso	May 2016–Jan 2020	11	8	1,189 (490–2,940)
	Kavuaya and Nkandu, DRC	Jan 2018–May 2020	51	30	348 (259–553)
	Sodo, Ethiopia	Jul 2017–Sep 2019	7	2	23 (10–67)
	Agogo, Ghana	May 2016–Apr 2019	60	10	112 (84–164)
	Imerintsiatosika, Madagascar	Feb 2016–Feb 2020	49	27	168 (135–233)
	Mahajanga, Madagascar	Jun 2018–Jan 2020	1	5	106 (9–710)
	Ibadan, Nigeria	Feb 2017–May 2020	65	1	42 (28–77)
GBD**	Eastern Mediterranean Region	2019	NA	NA	187 (118–281)
	Western Pacific Region		NA	NA	23 (15–33)
	Region of the Americas		NA	NA	3 (3–4)
	South-East Asia Region		NA	NA	306 (192–478)
	African Region		NA	NA	111 (71–166)
	European Region		NA	NA	2 (2–4)
	Global		NA	NA	119 (77–183)

**Abbreviations**: DRC = Democratic Republic of the Congo; GBD = Global Burden of Disease; NA = not available; SEAP = Surveillance for Enteric Fever in Asia Project; SETA = Severe Typhoid Fever in Africa; STRATAA = Strategic Typhoid Alliance Across Africa and Asia.

* Cases per 100,000 population.

^†^ https://doi.org/10.1016/S2214-109X(22)00119-X

^§^ https://doi.org/10.1016/S2214-109X(21)00370-3

^¶^ https://doi.org/10.2139/ssrn.4292849

** Global Burden of Disease Collaborative Network, GBD study, 2019. https://www.healthdata.org/gbd/gbd-2019-resources

**FIGURE:**
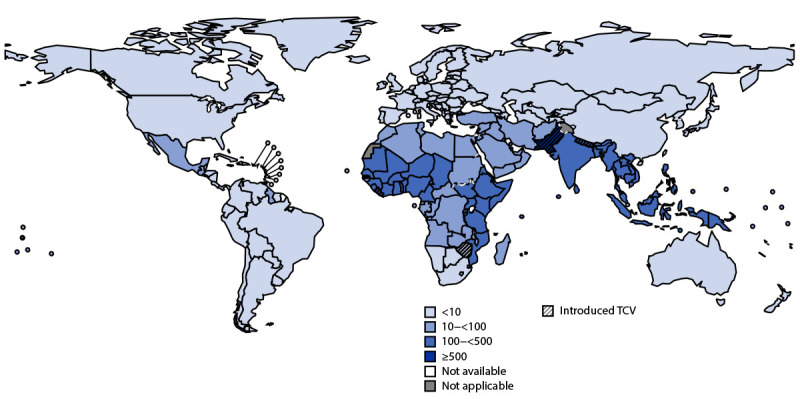
Estimated national typhoid fever incidence* and typhoid conjugate vaccine introduction^†^ status — worldwide, 2019 and 2022 **Source:** Global Burden of Disease Collaborative Network, Global Burden of Disease study, 2019. https://www.healthdata.org/gbd/gbd-2019-resources **Abbreviation: **TCV = typhoid conjugate vaccine. * Cases per 100,000 population. ^†^ Liberia, Nepal, Pakistan, Samoa, and Zimbabwe have introduced TCV.

An additional indication of typhoid fever burden can be obtained through analysis of outbreak^§§^ data. During 2017–2022, seven confirmed typhoid fever outbreaks were identified from ongoing outbreak monitoring activities by CDC’s Global Disease Detection Operation Center,^¶¶^ including the Philippines (2022: 14,056 cases) and three in Zimbabwe (January–March 2017: 1,312 cases; November 2017–February 2018: 3,187 cases; and August–December 2018: 7,134 cases), as well as outbreaks with confirmed antimicrobial-resistant cases in Pakistan (January 2018–December 2019: 14,894 cases) and China (2022: 23 cases) (*9*).

Apart from high disease incidence, the need for action is enhanced by the increasing prevalence of antimicrobial resistance in many countries with endemic typhoid fever. During 2010–2018, approximately 35% of reported *S*. Typhi isolates in Asia and 75% of those in Africa were resistant to chloramphenicol, ampicillin, and trimethoprim-sulfamethoxazole (defined as multidrug resistant [MDR]) (*10*). After a typhoid outbreak in Hyderabad, Pakistan in 2016, Pakistan became the first country to report MDR strains with additional resistance to fluoroquinolones and third-generation cephalosporins (defined as extensively drug resistant [XDR]); Pakistan continues to report high proportions of XDR *S*. Typhi cases (*2*). Resistance to an increasing number of antimicrobials, including fluoroquinolones, third-generation cephalosporins, and azithromycin (a macrolide), has been documented in Asia (*10*).

## Typhoid Conjugate Vaccine Introduction

WHO has prequalified two typhoid conjugate vaccines: Typbar-TCV (Bharat Biotech International Limited) and TYPHIBEV (Biological E. Limited)*** (*2*). Typhoid conjugate vaccines may be administered to persons aged ≥6 months, which facilitates their inclusion in routine immunization programs (*2*). A single dose administered to children has been shown to be safe and 79%–95% effective, with an antibody response persisting up to 7 years (*2*). Co-administration of typhoid conjugate vaccine with routinely administered vaccines (e.g., measles-containing vaccines, yellow fever vaccine, and serogroup A meningococcal conjugate vaccines) does not interfere with the immune response to typhoid conjugate vaccines or to the other simultaneously administered vaccines. Use of typhoid conjugate vaccine has been shown to be cost-effective for countries with high to very high typhoid fever incidence (*1*,*2*).

Since 2018, WHO has recommended that typhoid conjugate vaccine introduction be prioritized in countries with the highest typhoid fever incidence or a high prevalence of antimicrobial-resistant *S*. Typhi. Vaccine introduction should be implemented in combination with health education, WASH improvements, and health care worker training on typhoid fever diagnosis and treatment (*1*). The first public health introduction of typhoid conjugate vaccine occurred in 2018 in Navi Mumbai Municipal Corporation, India, as part of a program evaluation activity (*2*). Subsequently, typhoid conjugate vaccine has been introduced nationally into the routine immunization schedule for children at either age 9 months or 15–18 months in Pakistan (2019 Phase 1, 2021 Phase 2, and 2022 Phase 3), Liberia (2021), Zimbabwe (2021), Nepal (2022), and Samoa (2021 Phase 1 and 2022 Phase 2) (*4*) (Figure) (Table 2). Introduction in Malawi is planned for 2023.

**TABLE 2:** Typhoid conjugate vaccine introductions into routine immunization programs — worldwide, 2019–2022

Country	Program strategy	Targeted vaccination area*	Phase	Target population size^†^	Integrated health or other interventions	Catch-up campaign dates	Campaign status	Post campaign coverage, %^§^	Age at administration in routine program, mos
Pakistan	National, phased	Sindh	1	10,013,569	—	Nov 2019	Completed	82	9
		Punjab and Islamabad	2a	12,383,108	bOPV	Feb 2021	Completed	88 (Punjab) 69 (Islamabad)	
		Broader Punjab	2b	29,005,881	bOPV	Jun 2021	Completed	95	
		All other provinces	3	5,500,000	bOPV	Oct 2022	Completed	NA	
Liberia	National	—	—	1,900,000	—	Apr 2021	Completed	63	9
Zimbabwe	National	—	—	8,861,235	IPV, HPV, vitamin A	May 2021	Completed	NA	9
Samoa^¶^	National, phased	Upolu, Apia urban area	1	26,358	—	Aug–Sep 2021	Completed	84	9–12
			2	—	—	Ongoing	Ongoing	—	
Nepal	National	—	—	7,500,000	Hygiene education/ promotion and identification of under- and unvaccinated children	Apr–May 2022	Completed	NA	15

**Abbreviations:** bOPV = bivalent oral poliovirus vaccine; HPV = human papillomavirus vaccine; IPV = inactivated polio vaccine; NA = not available.

* If subnational.

^†^ Persons aged 9 months–14 years.

^§^ Post campaign coverage is based on immunization coverage survey.

^¶^ Country-financed introduction.

Catch-up vaccination campaigns targeting children aged 6 months–14 years are recommended at the time of introduction of typhoid conjugate vaccines into the routine immunization schedule, when feasible and supported by epidemiologic data, to maximize vaccination impact (*1*). Overall, more than 75 million children have received typhoid conjugate vaccines during catch-up campaigns, with post-campaign coverage estimates ranging from 63% to 95% (*2*). Nepal, Pakistan, and Zimbabwe conducted integrated campaigns that included other routine vaccines or identification of unvaccinated and undervaccinated children (*2*). Typhoid conjugate vaccine has also been used in outbreak response in Pakistan and Zimbabwe (*2*).

## Discussion

Since WHO recommended the use of typhoid conjugate vaccine to prevent typhoid fever in countries with endemic disease in 2018, only five countries, including three (7%) of the 44 countries and freely associated states with estimated high typhoid fever incidence based on Global Burden of Disease study estimates,^†††^ have introduced typhoid conjugate vaccines into their routine immunization schedule. Probable factors leading to delayed vaccine introduction include the presence of competing health priorities, particularly the COVID-19 pandemic, and insufficient disease burden data to guide national vaccine introduction decisions. Typhoid fever surveillance data are frequently limited to clinically suspected cases and serologic diagnostic tests with poor specificity. Population-based incidence studies are costly, time-consuming, technically challenging, and not available in most countries. Data on the prevalence of antimicrobial-resistant strains of *S*. Typhi are important for typhoid vaccine introduction decisions, but such data are lacking because of limited typhoid surveillance. Since 2018, additional data on the safety and effectiveness of typhoid conjugate vaccine and the lack of interference with other co-administered routine vaccines have become available and support the WHO typhoid vaccine introduction recommendation (*2*). Insufficient data from surveillance or population-based studies should not preclude considering typhoid conjugate vaccine introduction. Countries with endemic typhoid fever are encouraged to review regional and neighboring countries’ data, as well as national data sources such as published population-based studies, modeling data, laboratory-confirmed cases, antimicrobial testing studies, outbreak reports, and case reports of intestinal perforation (a hallmark of severe typhoid fever) to guide assessments of typhoid fever disease burden, and vaccine introduction decisions.

The five countries that have introduced typhoid conjugate vaccine have shared lessons learned regarding introduction strategies and integrated campaign opportunities. Among these five countries, Nepal, Pakistan, and Zimbabwe conducted integrated campaigns, including the simultaneous administration of other routine vaccines, vitamin A supplementation, hygiene promotion, or identification of undervaccinated children (*2*). Given the wide recommended age range for typhoid conjugate vaccine catch-up campaigns (6 months–14 years), school-based vaccination was found to be a useful strategy in Nepal, Pakistan, and Zimbabwe. However, drawbacks to such campaigns included difficulty reaching out-of-school children and increased absences on vaccination days, which schools ascribed to vaccine hesitancy stemming from misinformation related to the COVID-19 pandemic (*2*). Further country engagement is needed to better understand and address barriers to vaccination. Notably, four of the five countries that have introduced typhoid conjugate vaccine benefited from financial support from Gavi, the Vaccine Alliance (Gavi).^§§§^ Among the 44 countries considered to have high typhoid fever incidence, 11 middle-income countries are ineligible for Gavi support and might face financial barriers to typhoid conjugate vaccine introduction.

WHO recommends that countries with endemic typhoid fever establish and strengthen health care facility–based surveillance with laboratory confirmation, either through passive or active reporting to monitor disease trends and measure vaccine impact (*3*). Sentinel site surveillance has been critical for monitoring vaccine impact and disease trends for other vaccine-preventable diseases. Expanding blood culture diagnostic capacity strengthens surveillance for other invasive bacterial pathogens as well as typhoid fever and is integral to *S*. Typhi antimicrobial resistant strain surveillance. In addition, the development and validation of improved diagnostic tests and environmental surveillance might expand or augment typhoid surveillance in the future (*2*,*3*). In areas with endemic typhoid fever, nontraumatic intestinal perforation cases should be considered probable cases of typhoid or paratyphoid fever and have been used to identify outbreaks (*3*). Countries are encouraged to report laboratory-confirmed typhoid fever case data through eJRF to facilitate the monitoring of global typhoid fever incidence.

The findings in this report are subject to at least three limitations. First, data for both annual cases and outbreaks are underreported because of limited laboratory capacity. Second, the identification of typhoid fever outbreaks often relies on potentially incomplete reports from media, governments, or in-country technical partners including CDC and WHO; thus, outbreaks are likely underreported. Finally, recent programmatic experience with typhoid conjugate vaccine is still limited and accruing; therefore, data on routine typhoid conjugate vaccine coverage and its impact on disease are not yet available.

Use of typhoid conjugate vaccine in immunization programs is part of the multisectoral typhoid fever prevention approach, including WASH improvement and strengthened national surveillance, and will help countries reduce typhoid fever morbidity and mortality. Countries’ experiences with successful typhoid conjugate vaccine introductions and catch-up campaigns that included integrated health interventions could serve as examples for other countries planning to introduce typhoid conjugate vaccine. Sustained financial and technical commitment are needed at the national and international levels for improving WASH implementation, compiling national typhoid fever disease prevalence data, and increasing typhoid conjugate vaccination coverage to further advance typhoid fever control.

**Summary**

**What is already known about this topic?**

An estimated 11–21 million typhoid fever cases and 148,000–161,000 associated deaths occurred in 2015. The World Health Organization (WHO) recommends safe, effective typhoid conjugate vaccines (TCV) for typhoid fever control.

**What is added by this report?**

Population-based and modeling studies confirm high typhoid incidence in the WHO South-East Asian, Eastern Mediterranean, and African regions. Since 2018, five countries have introduced TCV into their national routine immunization schedule.

**What are the implications for public health practice?**

To guide evidence-based TCV introduction decisions, countries with endemic typhoid should consider all available information, including surveillance of laboratory-confirmed cases, population-based and modeling studies, and outbreak reports. Establishing and strengthening typhoid fever surveillance will be important to measure vaccine impact.

## Footnotes

* Consultant.

† World Bank gross national income (GNI) classification cutoffs per capita in U.S. dollars in 2021: high income >$12,695, upper-middle–income = $4,096–12,695, lower-middle–income = $1,046–4,095, and low income ≤$1,046. https://blogs.worldbank.org/opendata/new-world-bank-country-classifications-income-level-2021-2022

§ Countries with endemic disease are those where typhoid fever is common, typically low- and middle-income countries, and where much of the population lacks access to clean water, adequate sanitation, and standard hygiene.

¶ “Disease burden” is defined as an overall measure of the public health impact of typhoid fever on a given population. Disease burden can be measured as a composite of a variety of indicators, including morbidity (e.g., incidence and complications), mortality, and economic impact.

** Data on typhoid cases are reported by countries to WHO and UNICEF through eJRF and currently are not available online.

†† Incidence by region and age group is determined by Global Burden of Disease Collaborative Network, Global Burden of Disease study, 2019. https://www.healthdata.org/gbd/gbd-2019-resources

§§ Confirmed typhoid fever outbreaks are defined as an excess of suspected cases above what would normally be expected during a defined period for a defined community, geographic area, or season, with a minimum of two blood culture–confirmed cases.

¶¶ Event-based surveillance data collected during the time of the report might not reflect all cases, deaths, and reports of antimicrobial resistance associated with the stated outbreak. Outbreaks meeting criteria associated with International Health Regulations, Annex 2, are monitored by CDC’s Global Disease Detection Operation Center. https://www.cdc.gov/globalhealth/healthprotection/gddopscenter/index.html (Accessed January 6, 2023).

***
https://extranet.who.int/pqweb/vaccines

††† The 44 countries and freely associated states with high or very high incidence of typhoid fever, according to Global Burden of Disease 2019 estimates, are Bangladesh, Benin, Bhutan, Burkina Faso, Burma, Burundi, Cambodia, Cameroon, Chad, Côte d'Ivoire, Eritrea, Ethiopia, Gambia, Ghana, Guinea, India, Indonesia, Kenya, Laos, Liberia, Malaysia, Maldives, Mali, Mauritania, Mozambique, Nepal, Niger, Nigeria, Pakistan, Palau, Papua New Guinea, Philippines, Senegal, Seychelles, Sierra Leone, Somalia, South Sudan, Sri Lanka, Tanzania, Thailand, Timor-Leste, Togo, Uganda, and Vietnam.

§§§ In 2022, 54 countries were eligible for Gavi support because their average GNI per capita was ≤US $1,660 during the previous 3 years, based on World Bank data.
